# Some Points of the Histology of Malignant Tumors*Read before the Buffalo Microscopical Club, January 9, 1883.

**Published:** 1883-02

**Authors:** M. D. Mann

**Affiliations:** Professor of Obstetrics and Gynecology in University of Buffalo


### Some Points on the Histology of Malignant Tumors.*

By M. D. Mann, M. D.,

Professor of Obstetrics and Gynecology in University of Buffalo.

One of the advantages which has followed the introduction of the microscope into practical medicine is a more thorough understanding of the nature of the many new pathological formations which occur, from time to time, in the human body. These new growths, which we generally know under the name of tumors, while they have yielded some of the most brilliant triumphs to surgery, have, in the main, been somewhat vague and uncertain factors, or, at least, were so before the microscope turned its wondrous eye to the exploration of their hidden and complicated structure.

The discovery of the so-called cell doctrine was the necessary prelude to these researches, and it is to the originator of this doctrine, the great Virchow himself, whose name will always be honored as long as medicine is practiced as an art, or pursued as a science, that we owe the most valuable treatise ever written on the subject of tumors.

The point which has excited the greatest interest, and which has been most eagerly sought for, has been the discovery of some distinguishing mark by which, with the aid of the microscope, we should be able at once to distinguish those tumors having malignant characteristics from those which are benign.

From the great importance which, in the early stage of investigation, was given to the cell as a morphological unit, it was but natural that the mark of distinction should be sought in the varying characteristics of these bodies. So it soon became an accepted fact, with some pathologists, that cancerous tumors, for under this head were grouped all the malignant growths, contained certain cells, which, differing from cells formed in other tissues, were produced and found only in tumors of this class, and hence were characteristic of them.

*Read before the Buffalo Microscopical Club, January 9, 1883.

When brought to the test of experience, this theory broke down. Tumors which clinically showed the most marked malignancy did not contain a single cancer cell. It was shown, moreover, that the cells composing different varieties of malignant tumors belonged to different groups of cells, and that they had no close relationship one with another. So the doctrine of specific cell for cancer was given up, and the cancer-cell put in the long category of mistaken "facts," and is now delegated to the abode of myths. Still it haunts us, and we occasionally meet with allusions to it in the current literature of the day, which would seem to show that there were still some of its friends who had not heard of its demise.

With the fall of the cancer-cell there were other plans proposed for making easy the diagnosis of malignancy. One of the latest was that proposed by a surgeon who had dabbled a little in microscopy, but who had no adequate ideas of its limits. He proposed to extract a small portion of the tumor, and really invented a very ingenious harpoon for this purpose, and then, by examining the cells of the portion secured, and by determining their homologous or heterologous character as regards the tissues in which the tumors were situated, he expected to be able to determine the malignity or benignity of the growth in question. To aid us he gave a number of rules for the application of his method. But alas! when applied, these rules proved too much, for they showed that healthy granulation tissue was a malignant sarcoma, and a fœtus-in-uteri was a cancer.

How, then, can we determine the nature of a new growth? What are the microscopical appearances on which we may rely for a diagnosis? This question is often a practical and pressing one, both to the surgeon and to his patient. I will answer it, in the first place, by saying that as yet there is no decided and special mark which microscopically distinguishes a malignant from a benign growth. Is, then, the microscope of no use in this matter? Can the clinician gain no knowledge, no light, to help him in his difficult and often blind search after truth? To reply in the negative would be going too far; the truth lies between.

Let us see how the matter now stands. With the fall of the cancer-cell it was sought to determine some peculiarity in structure, which would separate the good from the bad tumors. But it soon became apparent that the line between malignancy and its opposite was a very difficult one to draw. It was found that tumors of a certain structure from one part of the body differed greatly in their clinical course from apparently similar structures in another part. It thus seemed evident that tumors must first be arranged in groups and classes, having a similar structure and that then, by long observation, the usual clinical course of each class must be determined as altered by seat, predisposition, age and many other conditions. This has now been done with a good deal of satisfaction as regards the more ordinary forms, but still leaving some rare and anomalous varieties, whose tendencies for good or for evil are in dispute.

The classes which we now recognize as malignant are the sarcomata and the carcinomata. While these two classes belong to the same group, as regards their malignancy, and also in structure, in that both are made up chiefly of cells, still they differ entirely as regards the nature of the cells of which they are composed, as well as in the arrangement of their ultimate elements.

The sarcomas belong to the connective-tissue group, and are composed of cells which resemble some one of the different cells which belong to this group. As a type they have embryonic connective-tissue. The cells are round, either large or small, with large nuclei or small, as the case may be; or they are fusiform or stellate, according to the member of the histological connective-tissue group in which they had their origin, or which they simulate. Thus we find sarcoma simulating cartilage chondro-sarcoma, also osteo-sarcoma, from bone; glio-sarcoma, from neuralgia. Again, the sarcoma may be mixed with other elements, forming a class of mixed tumors whose exact place in the scale of malignancy it is very hard to determine.

But the recognition of the presence of sarcomatous tissue in a given tumor is at times very difficult, and is largely a question of judgment and must be decided only after a careful study of all the features of the case, clinically as well as histologically. For instance, a fibroma of the ear can hardly be distinguished, microscopically, from a fibro-sarcoma, taken from another part of the body. The history of the case, however, makes it clear at once. Again, the line between a simple chondroma and chondro-sarcoma, is sometimes very difficult to draw. The distinction lies in the number of the cells, the sarcoma being much better supplied in this respect than its type-tumors, the simple cartilage tumor. But to say just when the cells become numerous enough to warrant us in calling it a sarcoma, is a very nice point to decide. Here again the history of the case, the rate of development and the age of the patient, the presence or absence of other tumors, all come in to aid us in forming a just opinion and in hazarding a prediction as to its future course. Of all the points just mentioned, the rate of growth is the most prominent and important.

I well remember where the diagnosis lay between sarcoma and granulation tissue in a very obscure and doubtful case. The microscope was hardly able to decide definitely, but the fact that the tumor had been very slow in its growth and the ultimate discovery of a little pus in connection with it, decided the matter in favor of the inflammatory theory and the result justified the diagnosis. This leads me to a few further remarks as to the histology of these tumors. Composed of connective-tissue cells of some sort, the cells are held together by a distinct intercellular substance. This may be like the intercellular substance of cartilage or bone, or may be simply an almost undemonstrably fine reticulum of fibres and basement-substance.

Considerable importance must be placed on the number of cells as relative to the amount of intercellular substance. The greater the number of cells, as a rule, the greater the malignancy and the more rapid the growth. One peculiarity is the arrangement of the vessels. There are very few true vessels, the blood being carried through the tissues in channels which have either very delicate and thin walls or are more generally mere channels. It is this peculiar arrangement of the vessels which accounts for the readiness with which these tumors bleed, and it also explains the tendency to the formation of metastatic tumors in other places. These secondary tumors are formed by the breaking off of a tissue, and its conveyance by the blood stream into the general circulation. Here it is carried to the lungs, liver or some of the internal organs whose extremely delicate capillaries will not admit of its further passage; being thus fixed, the cells imbibe nourishment from the surrounding blood, and thus become the focus for new growths.

So much for the sarcomas. If they present difficulties, how is it with their near relatives, the carcinomata?

These tumors are justly described as being epithelial in their nature. If we adopt the theories of Waldyer, Bilroth, and their fellow-workers, and decide that no tissues are indifferent, but that like is only produced by like, we must suppose that a carcinoma can only originate in a tissue having epithelial elements. The name was formerly used, like the English term cancer, to designate any form of malignant tumor. But now that we understand better the histology of these functions, we limit its application to those tumors having cells like the epithelial cells. But besides the epithelial cells there are other elements. There is a distinct connective tissue stroma, and it is in the cavities of this stroma that the cells are found, while the vessels run in its substance. The cells are huddled together, often without any attempt at arrangement, and without intercellular substance. But this general plan of arrangement is often modified. In one variety of carcinoma, which we distinguish by the unfortunate name of epithelioma, we often find a distinct arrangement of the cells in tubular follicles, or covering tufts. The cells also resemble, very closely, the tissue from which they spring, be they large or small pavement epithelium or the familiar cylindrical cells, such as are found so plentifully in the gastro-intestinal tract. The flat cells are often closely packed into concentric nests or pearls, an appearance which was long thought to be characteristic of this form of growth, an opinion, however, which is now acknowledged to be a mistake, they having been found in other growths and under other conditions. It is in this division of the carcinoma that we find the greatest difficulty in making a diagnosis. They trend so closely, on the one side, towards the adenomata or glandular tumors, and on the other to what have been called the true carcinomata, that the line between them is very hard to draw. Here, again, the history of the case, the seat of the tumor, the rapidity of its growth, all come to our aid, and are simply indispensable in enabling us to make a prognosis.

The true carcinoma consists, like the last considered variety, of connective-tissue stroma and of cells. The stroma is either dense and abundant or scanty and soft. In the first instance it is called scirrhus and in the second a medullary or encephaloid growth. The cells are of an irregular type, with large and distinct nuclei. They can be recognized as being of an epithelial type, but they do not exactly resemble normal epithelium, and are often malformed and misshapen. It is to this rapidly growing epithelium that we owe the myth of the cancer-cell, and we are now in a position to see how it came to have its original position, and the cause of its downfall.

Thus far, while describing to you some points in the histology of malignant tumors, I have, as it were, been entering a plea for the microscope, and for a proper understanding of its true position in reference to the very important matter, the diagnosis of tumors.

20

A great deal of misunderstanding exists at present in the mind of the medical profession, and only a few days since I saw the remark of a distinguished clinician that he had ceased to place any dependence on the microscope as a means of diagnosis. As I have already pointed out, there is no absolute mark by which a malignant tumor may be distinguished from a benign, and in a difficult case an immense amount of experience and knowledge is necessary on the part of the microscopist to render his opinion of any value, and when he has given it, it is, after all, only an opinion and not an absolute dictum.

The sooner this is understood the better for the credit of our instrument, the sooner will its true value be appreciated and understood. Too much reliance has been placed on its revelations. It has been thought by the uninitiated that we would see, with our lenses, the word "cancer" printed on every cell and every fibre, and that all that were necessary was to have a small bit of a suspected growth to enable us to say absolutely as to its character.

I have pointed out to you this mistake and have insisted that the microscopical appearances are to be considered as only one factor, a very important factor to be sure, but still only one among many in forming our opinion as to the true nature of a suspected tumor. That experience and skill are very important on the part of the microscopist, and that only in well marked cases is his opinion to be placed in advance of that formed by the careful and experienced clinical observer.

